# CANNABIS USE AMONG INFORMAL CAREGIVERS OVER 50: FINDINGS FROM THE CALIFORNIA HEALTH INTERVIEW SURVEY

**DOI:** 10.1093/geroni/igae098.1244

**Published:** 2024-12-31

**Authors:** Fadi Martinos, Divya Bhagianadh

**Affiliations:** University of Iowa, Iowa City, Iowa, United States; University of Arkansas, Fayetteville, Arkansas, United States

## Abstract

Despite the availability of evidence-based therapeutics and supports, informal primary care partners of older adults are using cannabis as an unproven, potentially harmful, alternative for reducing physical burden and psychological distress. Using the 2019 California Health Interview Survey, we identified 2,278 respondents over 50 years old who indicated providing care to someone also over 50 in the past year. We determined 23.0% of these survey respondents between 50 to 64 years and 18.0% of those over 65 years older used cannabis in the past year, and older caregivers were more likely to use cannabis than non-caregivers. Cannabis use among older caregivers was associated with smoking and self-reported feelings of depression (AOR 1.76) and/or nervousness (AOR 2.06). Cannabis use among older caregivers was not associated with the burden of caregiving or the condition of the care recipient. Caregivers who use cannabis were more likely to experience a delay in accessing health care services relative to caregivers who do not use (AOR1.54).However, they were no more or less likely to use emergency department or outpatient medical care. These findings illuminate how cannabis is being used as a understudied form of self-care among informal care partners.

